# β-Li_0.37_Na_0.63_Fe(MoO_4_)_2_

**DOI:** 10.1107/S1600536814000646

**Published:** 2014-01-18

**Authors:** Amira Souilem, Mohamed Faouzi Zid, Ahmed Driss

**Affiliations:** a Laboratoire de Matériaux et Cristallochimie, Faculté des Sciences de Tunis, Université de Tunis ElManar, 2092 Manar II Tunis, Tunisia

## Abstract

The title compound, lithium/sodium iron(III) bis­[ortho­molyb­date(VI)], was obtained by a solid-state reaction. The main structure units are an FeO_6_ octa­hedron, a distorted MoO_6_ octa­hedron and an MoO_4_ tetra­hedron sharing corners. The crystal structure is composed of infinite double MoFeO_11_ chains along the *b-*axis direction linked by corner-sharing to MoO_4_ tetra­hedra so as to form Fe_2_Mo_3_O_19_ ribbons. The cohesion between ribbons *via* mixed Mo—O—Fe bridges leads to layers arranged parallel to the *bc* plane. Adjacent layers are linked by corners shared between MoO_4_ tetra­hedra of one layer and FeO_6_ octa­hedra of the other layer. The Na^+^ and Li^+^ ions partially occupy the same general position, with a site-occupancy ratio of 0.631 (9):0.369 (1). A comparison is made with *A*Fe(MoO_4_)_2_ (*A* = Li, Na, K and Cs) structures.

## Related literature   

For the electrochemical performance of similar materials, see: Padhi *et al.* (1997 ▶); Prakash *et al.* (2000 ▶); Okuyama *et al.* (2001 ▶); Croce *et al.* (2003 ▶). For their physical properties, see: Nagpure *et al.* (2010 ▶); Prasad & Varma (1994 ▶); Chen *et al.* (2009 ▶); Tomohiro *et al.* (2002 ▶). For related structures, see: van der Lee *et al.* (2008 ▶); Klevtsova (1975 ▶); Baza­rov *et al.* (2010 ▶). For bond-valence parameters, see: Brown & Altermatt, (1985 ▶).

## Experimental   

### 

#### Crystal data  

Li_0.37_Na_0.63_Fe(MoO_4_)_2_
*M* *_r_* = 392.78
Triclinic, 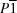

*a* = 6.8860 (8) Å
*b* = 7.2560 (9) Å
*c* = 7.3786 (9) Å
α = 91.060 (6)°
β = 110.933 (9)°
γ = 105.661 (9)°
*V* = 328.83 (7) Å^3^
*Z* = 2
Mo *K*α radiation
μ = 5.98 mm^−1^
*T* = 298 K
0.28 × 0.21 × 0.14 mm

#### Data collection  

Enraf-Nonius CAD-4 diffractometer
Absorption correction: ψ scan (North *et al.*, 1968 ▶) *T*_min_ = 0.286, *T*_max_ = 0.488
2838 measured reflections
1431 independent reflections
1380 reflections with *I* > 2σ(*I*)
*R*_int_ = 0.015
2 standard reflections every 120 min intensity decay: 1.3%

#### Refinement  

*R*[*F* ^2^ > 2σ(*F* ^2^)] = 0.020
*wR*(*F* ^2^) = 0.051
*S* = 1.19
1431 reflections
112 parameters
Δρ_max_ = 1.23 e Å^−3^
Δρ_min_ = −0.58 e Å^−3^

### 

Data collection: *CAD-4 EXPRESS* (Duisenberg, 1992 ▶; Macíček & Yordanov, 1992 ▶); cell refinement: *CAD-4 EXPRESS*; data reduction: *XCAD4* (Harms & Wocadlo, 1995 ▶); program(s) used to solve structure: *SHELXS97* (Sheldrick, 2008 ▶); program(s) used to refine structure: *SHELXL97* (Sheldrick, 2008 ▶); molecular graphics: *DIAMOND* (Brandenburg & Putz, 1999 ▶); software used to prepare material for publication: *WinGX* (Farrugia, 2012 ▶).

## Supplementary Material

Crystal structure: contains datablock(s) I. DOI: 10.1107/S1600536814000646/vn2079sup1.cif

Structure factors: contains datablock(s) I. DOI: 10.1107/S1600536814000646/vn2079Isup2.hkl

CCDC reference:

Additional supporting information:  crystallographic information; 3D view; checkCIF report

## Figures and Tables

**Table 1:** Selected bond lengths (Å)

Mo1—O5	1.737 (3)
Mo1—O6	1.768 (3)
Mo1—O1^i^	1.775 (2)
Mo1—O2^ii^	1.787 (2)
Mo2—O4	1.701 (3)
Mo2—O7	1.707 (3)
Mo2—O8	1.880 (2)
Mo2—O3	1.884 (2)
Mo2—O3^iii^	2.400 (3)
Mo2—O5	2.636 (3)
Fe1—O6	1.947 (3)
Fe1—O8^iv^	1.961 (2)
Fe1—O3^v^	1.975 (2)
Fe1—O2^v^	2.002 (2)
Fe1—O8^vi^	2.041 (2)
Fe1—O1^v^	2.101 (2)
Na1—O7^vii^	2.123 (4)
Na1—O5^v^	2.211 (3)
Na1—O4	2.252 (4)
Na1—O1^v^	2.286 (3)
Na1—O3^v^	2.306 (3)
Na1—O7^vi^	2.718 (4)

Symmetry codes: (i) 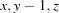; (ii) 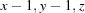; (iii) 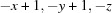; (iv) 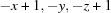; (v) 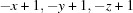; (vi) 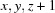; (vii) 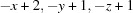.

**Fig. 1.**
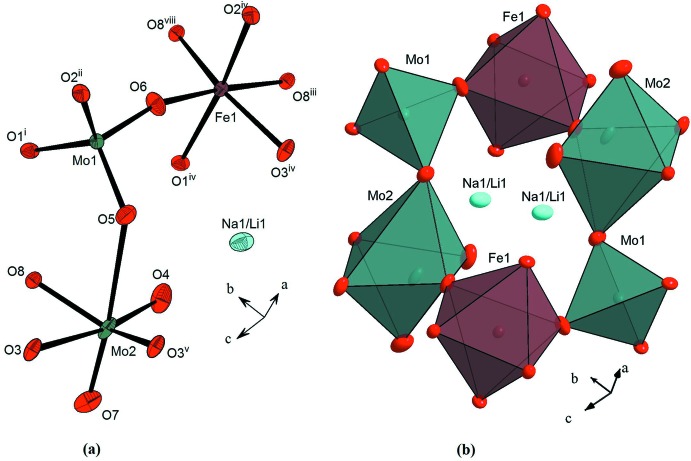
(a): Unité asymétrique dans le composé Li0.37Na0.63FeMo2O8. Les éllipsoïdes ont été définis avec 50% de probabilité, (b): Nouvelle unité centrosymétrique. [Symmetry codes: (i) 1 - x,2 - y,1 - z; (ii) 2 - x,2 - y,1 - z; (iii) 1 - x,1 - y,1 - z; (iv) x,y,z; (v) 1 - x,1 - y,1 - z; (viii) x,1 + y,z].

**Fig. 2.**
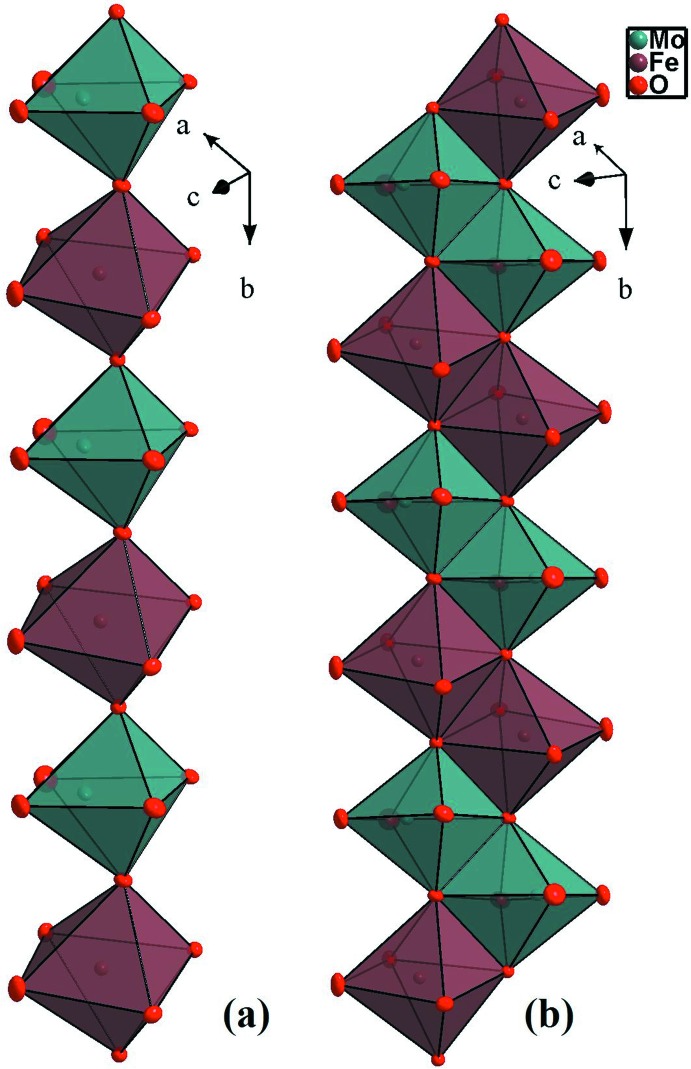
(a): Représentation d'une chaîne simple formée par les octaèdres MO6 (M=Mo, Fe), (b): Représentation d'une chaîne double.

**Fig. 3.**
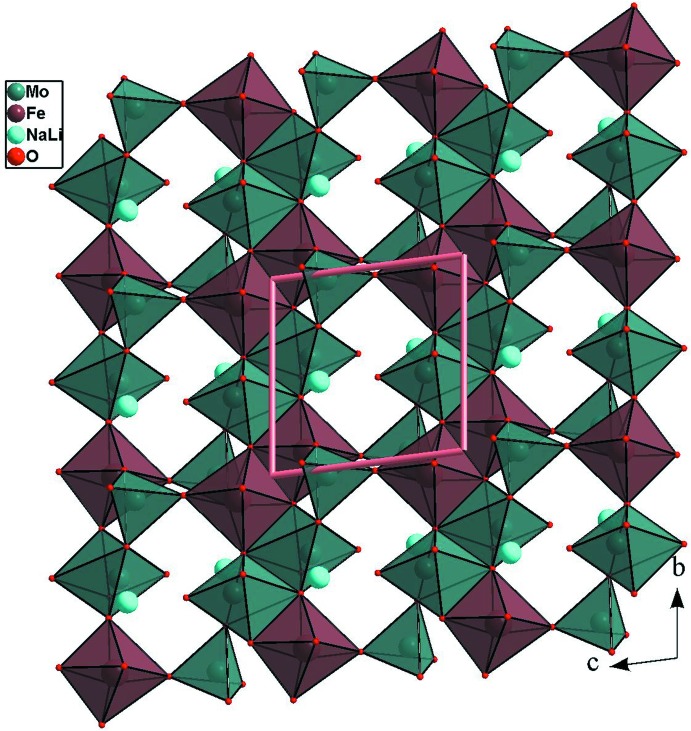
Projection de la couche selon le plan bc mettant en évidence la jonction des rubans.

**Fig. 4.**
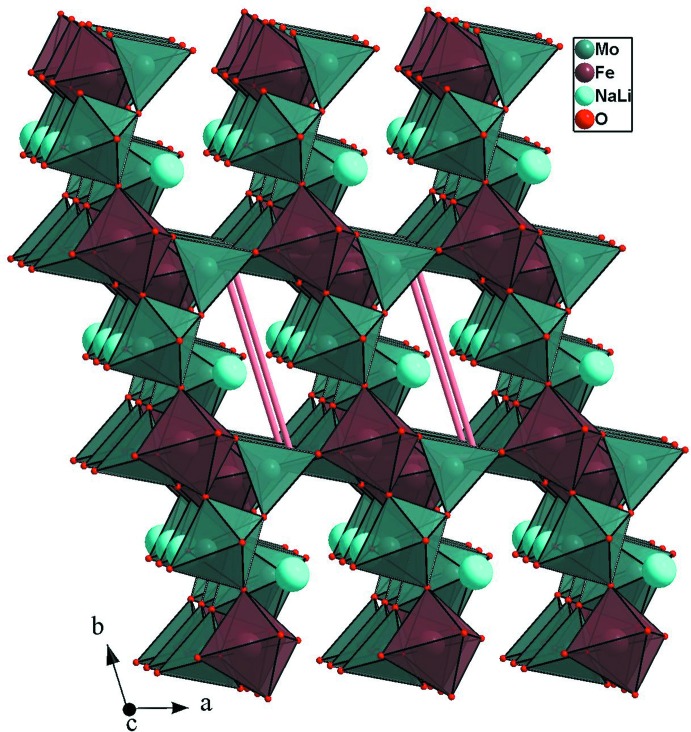
Projection de la structure de Li0.37Na0.63FeMo2O8 selon c montrant la jonction entre couches.

**Fig. 5.**
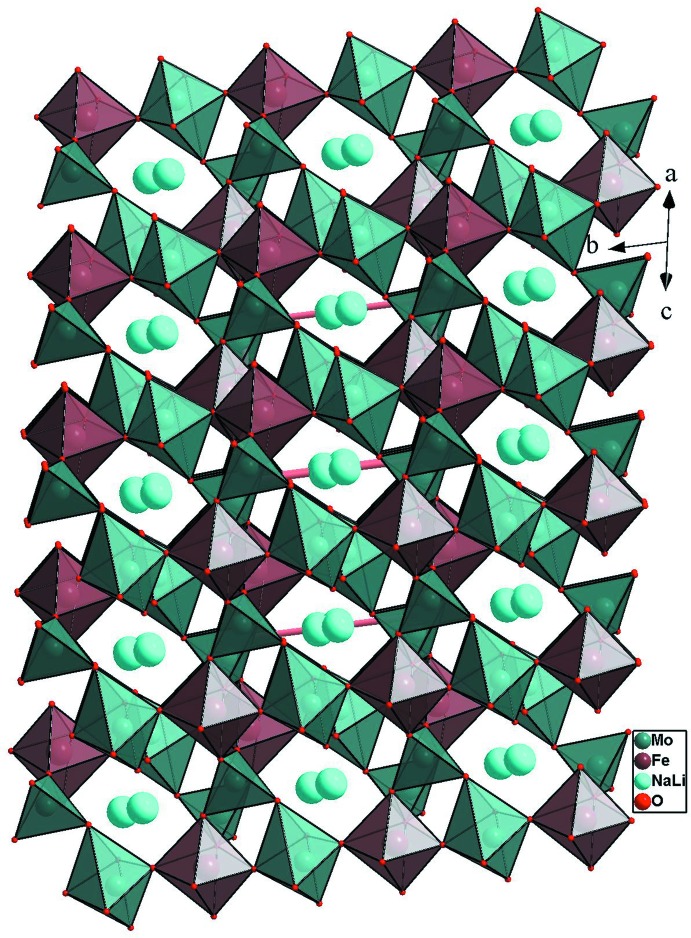
Projection de la structure de Li0.37Na0.63FeMo2O8 selon [101] montrant les tunnels où résident les cations.

**Fig. 6.**
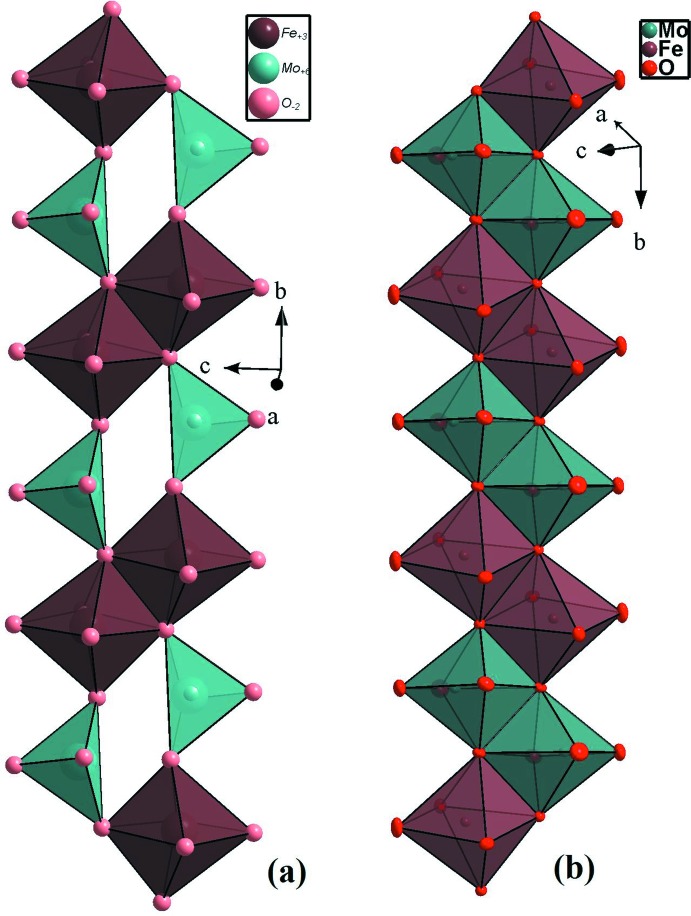
Jonction des polyèdres dans la structure de, (a): LiFe(MoO4)2 et (b): Li0.37Na0.63FeMo2O8.

## supplementary crystallographic information

### 1. Comment

La révolution technologique de ces dernières années a donné vie aux batteries rechargeables au lithium (LiFePO_4_) (Padhi *et al.*, 1997) qui sont un dispositif important pour le stockage de l'énergie électrique. L'avantage de l'utilisation du lithium est qu'il possède le potentiel électrochimique le plus élevé par rapport à l'électrode à hydrogène standard ce qui confère à la batterie une plus haute tension.

Les ressources terrestres limitées en lithium ainsi que son cout noté assez cher nous a incité à substituer ce dernier par le sodium vu son abondance et son faible cout (les batteries rechargeables au sodium (Na-NiCl2) et sodium-soufre) (Prakash *et al.*, 2000; Okuyama *et al.*, 2001). De plus le remplacement de l'élément Ni par le molybdène, a permis d'améliorer les performances électrochimiques de ces matériaux (Croce *et al.*, 2003).

Afin de suivre cette démarche de développement, ce travail est une contribution à l'étude des composés appartenant à la grande famille des molybdates de fer, de métaux alcalins et pseudo-alcalins. Ils possèdent plusieurs domaines d'applications notamment catalyse, électriques, ferroélectriques, magnétiques (Nagpure *et al.*, 2010; Prasad & Varma, 1994; Chen *et al.*, 2009; Tomohiro *et al.*, 2002).

En effet, une nouvelle phase de formulation Li_0.37_Na_0.63_FeMo_2_O_8_ a été obtenue par voie sèche. L'unité asymétrique est formée d'un octaèdre FeO_6_, un second de type MoO_6_ et d'un tétraèdre MoO_4_ (Fig. 1a). Deux unités formulaires centrosymétriques se regroupent par partage de sommets donnant lieu à un dimère cyclique (Fig. 1b). La structure peut être décrite au moyen de chaînes classiques MoFeO_11_, de type MO_3_ (*M* = W, Mo, V, Cr, Fe, Ti), formées par des octaèdres MoO_6_ et FeO_6_ partageant que des sommets (Fig. 2a). Chaque chaîne se regroupe, par mise en commun d'arêtes entre octaèdre de même natue, avec son adjacente pour conduire à des doubles chaînes (Fig. 2 b) similaires à celles rencontrées dans la série d'oxydes A*MM*'O_6_ (*M*' = V, Mn, Cr). Les tétraèdre MoO_4_ se lient par partage de sommets aux doubles chaînes pour former des rubans de type Fe_2_Mo_3_O_19_ (Fig. 3). Ces derniers se regroupent par formation de ponts mixtes Mo–O–Fe entre tétraèdres MoO_4_ et octaèdres FeO_6_ pour conduire à des couches disposées parallèlement aux plans *bc* (Fig. 3). La jonction de ses dernières par partage de sommets entre tétraèdres MoO_4_ et octaèdres FeO_6_ appartenant respectivement à deux couches adjacentes conduit à une charpente tridimensionnelle (Fig. 4). La projection de la structure du composé Li_0.37_Na_0.63_FeMo_2_O_8_ selon la direction [101] révèle la présence de canaux où résident les cations Li^+^ et Na^+^ (Fig. 5).

L'examen des facteurs géométriques dans la structure montre qu'ils sont en bon accord avec ceux rencontrés dans la littérature (van der Lee *et al.*, 2008; Klevtsova 1975; Bazarov *et al.*, 2010).

D'autre part, le calcul des valences de liaisons (BVS), utilisant la formule empirique de Brown (Brown & Altermatt, 1985), conduit aux valeurs des charges des cations suivants: Mo1(5,919), Mo2(6,033), Fe1(3,059) et (Na/Li)(1,184).

Un examen rigoureux de différentes structures trouvées dans la littérature révèle que le matériau étudié est un nouveau membre d'une famille de formule *A*FeMo_2_O_8_ (*A*= Na, Li, K, Cs) incluant NaFe(MoO_4_)_2_ (Klevtsova, 1975), LiFe(MoO_4_)_2_ (van der Lee *et al.*, 2008) et CsFe(MoO_4_)_2_ (Bazarov *et al.*, 2010).

Les structures des molybdates de fer ayant comme groupe d'espace *P*-3*m* (Bazarov *et al.*, 2010) ou *C*2/*c* (Klevtsova, 1975), ont le même type de couches où le molybdène occupe seulement les sites tétraédriques. Dans le cas des composés ayant une formulation analogue de type *A*Fe(MoO_4_)_2_ cristallisant dans le système triclinique, on peut rencontrer le molybdène occupant totalement les sites tétraédriques (*A* = Li; van der Lee *et al.*, 2008) (forme α) donnant naissance à de nouveaux types de couches. Dans le composé étudié, Li_0.37_Na_0.63_FeMo_2_O_8_ (forme β), le molybdène occupe les sites tétraédriques et aussi octaédriques. Cette différence dans l'occupation des sites a donné lieu à de nouveaux types de doubles chaînes (Fig. 6).

### 2. Experimental

La synthèse de Li_0.37_Na_0.63_FeMo_2_O_8_ a été effectuée par réaction à l'état solide, à partir d'un mélange de carbonate de sodium (FLUKA, 71350), carbonate de lithium (AZIENDA CHIMICA, 104094819), de nitrate de fer (FLUKA 44949) et de molybdate d'ammonium (FLUKA, 69858) dans des rapports molaires Li:Na:Fe:Mo respectivement égaux à 1:3:4:8. Après un broyage poussé dans un mortier en agate, le mélange est placé dans un creuset en porcelaine, puis porté dans un premier temps à une température de 673 K pendant 4 heures, en vue d'éliminer les produits volatils. Un second traitement thermique a été effectué à la température de synthèse proche de la fusion, 1143 K pendant 72 heures. Le résidu final a subi ensuite un refroidissement lent de (5°/12 h) jusqu'à 1043 K, suivi d'un autre plus rapide (50°/24 h) jusqu'à la température ambiante. Des cristaux de couleur verdâtre sont séparés à l'eau bouillante.

### 3. Refinement

L'utilisation des fonctions SUMP et EADP autorisées par le programme *SHELX*, pour les ions Na1 et Li1 conduit à des ellipsoïdes bien définis. De plus, les densités d'électrons maximum et minimum restants dans la Fourier-différence sont acceptables et sont situées respectivements à 0.85 Å de O6 et à 0.80 Å de Mo1.

L'affinement a été réalisé en considérant les atomes d'oxygène O5 et O3 dans la sphère de coordination de d'atome de Molybdène Mo2. En effet le calcul des valences de charges utilisant la formule de Brown (Brown & Altermatt (1985)), montre que ces dernières sont égales à 0.2853 v.u. et 0.1559 v.u. respectivement pour O3 et O5. Elles sont donc considérées non négligeables et ces deux atomes peuvent par conséquent compléter l'environnement octaèdrique (*d*(Mo2–O5^iv^)= 2.600 (3) Å) de l'atome Mo2 et sa charge (+6.033).

### Crystal data

**Table:**

Li_0.37_Na_0.63_Fe(MoO_4_)_2_	*Z* = 2
*M**_r_* = 392.78	*F*(000) = 364
Triclinic, *P*1	*D*_x_ = 3.967 Mg m^−^^3^
Hall symbol: -P 1	Mo *K*α radiation, λ = 0.71073 Å
*a* = 6.8860 (8) Å	Cell parameters from 25 reflections
*b* = 7.2560 (9) Å	θ = 11–15°
*c* = 7.3786 (9) Å	µ = 5.98 mm^−^^1^
α = 91.060 (6)°	*T* = 298 K
β = 110.933 (9)°	Prism, green
γ = 105.661 (9)°	0.28 × 0.21 × 0.14 mm
*V* = 328.83 (7) Å^3^	

### Data collection

**Table:**

Enraf-Nonius CAD-4 diffractometer	1380 reflections with *I* > 2σ(*I*)
Radiation source: fine-focus sealed tube	*R*_int_ = 0.015
Graphite monochromator	θ_max_ = 27.0°, θ_min_ = 2.9°
ω/2θ scans	*h* = −8→8
Absorption correction: ψ scan (North *et al.*, 1968)	*k* = −9→9
*T*_min_ = 0.286, *T*_max_ = 0.488	*l* = −9→9
2838 measured reflections	2 standard reflections every 120 min
1431 independent reflections	intensity decay: 1.3%

### Refinement

**Table:**

Refinement on *F*^2^	Primary atom site location: structure-invariant direct methods
Least-squares matrix: full	Secondary atom site location: difference Fourier map
*R*[*F*^2^ > 2σ(*F*^2^)] = 0.020	*w* = 1/[σ^2^(*F*_o_^2^) + (0.021*P*)^2^ + 0.8764*P*] where *P* = (*F*_o_^2^ + 2*F*_c_^2^)/3
*wR*(*F*^2^) = 0.051	(Δ/σ)_max_ < 0.001
*S* = 1.19	Δρ_max_ = 1.23 e Å^−^^3^
1431 reflections	Δρ_min_ = −0.58 e Å^−^^3^
112 parameters	Extinction correction: *SHELXL97* (Sheldrick, 2008), Fc^*^=kFc[1+0.001xFc^2^λ^3^/sin(2θ)]^-1/4^
0 restraints	Extinction coefficient: 0.0119 (9)

### Special details

**Table:**

Geometry. All e.s.d.'s (except the e.s.d. in the dihedral angle between two l.s. planes) are estimated using the full covariance matrix. The cell e.s.d.'s are taken into account individually in the estimation of e.s.d.'s in distances, angles and torsion angles; correlations between e.s.d.'s in cell parameters are only used when they are defined by crystal symmetry. An approximate (isotropic) treatment of cell e.s.d.'s is used for estimating e.s.d.'s involving l.s. planes.
Refinement. Refinement of *F*^2^ against ALL reflections. The weighted *R*-factor *wR* and goodness of fit *S* are based on *F*^2^, conventional *R*-factors *R* are based on *F*, with *F* set to zero for negative *F*^2^. The threshold expression of *F*^2^ > σ(*F*^2^) is used only for calculating *R*-factors(gt) *etc*. and is not relevant to the choice of reflections for refinement. *R*-factors based on *F*^2^ are statistically about twice as large as those based on *F*, and *R*- factors based on ALL data will be even larger.

### Fractional atomic coordinates and isotropic or equivalent isotropic displacement parameters (Å^2^)

**Table:**

	*x*	*y*	*z*	*U*_iso_*/*U*_eq_	Occ. (<1)
Mo1	0.18308 (4)	0.03418 (4)	0.28342 (4)	0.00999 (11)	
Mo2	0.66625 (5)	0.42440 (4)	0.20010 (5)	0.01552 (11)	
Fe1	0.39452 (8)	0.09553 (7)	0.81999 (7)	0.00988 (13)	
Na1	0.7648 (4)	0.4490 (3)	0.7567 (4)	0.0221 (8)	0.631 (9)
Li1	0.7648 (4)	0.4490 (3)	0.7567 (4)	0.0221 (8)	0.369 (9)
O1	0.3163 (4)	0.8758 (3)	0.2278 (4)	0.0137 (5)	
O2	0.8946 (4)	0.9550 (4)	0.1547 (4)	0.0145 (5)	
O3	0.5285 (4)	0.6200 (3)	0.1475 (4)	0.0171 (5)	
O4	0.7530 (5)	0.4334 (4)	0.4476 (4)	0.0267 (6)	
O5	0.2937 (4)	0.2661 (4)	0.2401 (4)	0.0203 (6)	
O6	0.2403 (4)	0.0424 (4)	0.5373 (4)	0.0205 (6)	
O7	0.8963 (5)	0.5147 (4)	0.1521 (5)	0.0284 (7)	
O8	0.5823 (4)	0.1597 (3)	0.1114 (3)	0.0118 (5)	

### Atomic displacement parameters (Å^2^)

**Table:**

	*U*^11^	*U*^22^	*U*^33^	*U*^12^	*U*^13^	*U*^23^
Mo1	0.00649 (16)	0.01197 (16)	0.01053 (16)	0.00259 (11)	0.00226 (11)	−0.00055 (10)
Mo2	0.01547 (17)	0.00794 (16)	0.01610 (17)	0.00378 (12)	−0.00234 (12)	−0.00076 (11)
Fe1	0.0090 (2)	0.0088 (2)	0.0107 (2)	0.00255 (17)	0.00243 (18)	0.00021 (17)
Na1	0.0196 (12)	0.0174 (12)	0.0338 (14)	0.0070 (9)	0.0138 (10)	0.0076 (9)
Li1	0.0196 (12)	0.0174 (12)	0.0338 (14)	0.0070 (9)	0.0138 (10)	0.0076 (9)
O1	0.0108 (11)	0.0136 (12)	0.0192 (13)	0.0043 (9)	0.0079 (10)	0.0032 (10)
O2	0.0079 (11)	0.0197 (13)	0.0160 (12)	0.0042 (9)	0.0046 (9)	−0.0008 (10)
O3	0.0194 (13)	0.0091 (11)	0.0184 (13)	0.0053 (10)	0.0015 (10)	−0.0009 (9)
O4	0.0291 (15)	0.0247 (15)	0.0164 (14)	0.0096 (12)	−0.0039 (11)	−0.0030 (11)
O5	0.0191 (13)	0.0144 (12)	0.0253 (14)	0.0021 (10)	0.0080 (11)	−0.0002 (11)
O6	0.0158 (13)	0.0329 (15)	0.0112 (12)	0.0074 (11)	0.0034 (10)	−0.0008 (11)
O7	0.0238 (15)	0.0207 (14)	0.0334 (17)	0.0006 (12)	0.0069 (13)	0.0042 (12)
O8	0.0124 (11)	0.0097 (11)	0.0119 (11)	0.0034 (9)	0.0028 (9)	0.0014 (9)

### Geometric parameters (Å, º)

**Table:**

Mo1—O5	1.737 (3)	Fe1—O8^iv^	1.961 (2)
Mo1—O6	1.768 (3)	Fe1—O3^v^	1.975 (2)
Mo1—O1^i^	1.775 (2)	Fe1—O2^v^	2.002 (2)
Mo1—O2^ii^	1.787 (2)	Fe1—O8^vi^	2.041 (2)
Mo2—O4	1.701 (3)	Fe1—O1^v^	2.101 (2)
Mo2—O7	1.707 (3)	Na1—O7^vii^	2.123 (4)
Mo2—O8	1.880 (2)	Na1—O5^v^	2.211 (3)
Mo2—O3	1.884 (2)	Na1—O4	2.252 (4)
Mo2—O3^iii^	2.400 (3)	Na1—O1^v^	2.286 (3)
Mo2—O5	2.636 (3)	Na1—O3^v^	2.306 (3)
Fe1—O6	1.947 (3)	Na1—O7^vi^	2.718 (4)
			
O5—Mo1—O6	107.29 (13)	O6—Fe1—O8^iv^	101.81 (11)
O5—Mo1—O1^i^	110.16 (12)	O6—Fe1—O3^v^	99.65 (12)
O6—Mo1—O1^i^	106.37 (12)	O8^iv^—Fe1—O3^v^	156.83 (11)
O5—Mo1—O2^ii^	110.72 (12)	O6—Fe1—O2^v^	88.11 (11)
O6—Mo1—O2^ii^	108.81 (12)	O8^iv^—Fe1—O2^v^	92.41 (10)
O1^i^—Mo1—O2^ii^	113.22 (11)	O3^v^—Fe1—O2^v^	96.92 (11)
O4—Mo2—O7	105.39 (15)	O6—Fe1—O8^vi^	174.67 (11)
O4—Mo2—O8	103.81 (12)	O8^iv^—Fe1—O8^vi^	78.88 (11)
O7—Mo2—O8	103.08 (13)	O3^v^—Fe1—O8^vi^	78.95 (10)
O4—Mo2—O3	103.37 (13)	O2^v^—Fe1—O8^vi^	97.16 (10)
O7—Mo2—O3	103.71 (13)	O6—Fe1—O1^v^	87.72 (11)
O8—Mo2—O3	134.72 (11)	O8^iv^—Fe1—O1^v^	84.61 (10)
O4—Mo2—O3^iii^	168.20 (13)	O3^v^—Fe1—O1^v^	87.65 (10)
O7—Mo2—O3^iii^	86.38 (13)	O2^v^—Fe1—O1^v^	174.27 (10)
O8—Mo2—O3^iii^	72.08 (9)	O8^vi^—Fe1—O1^v^	87.08 (10)
O3—Mo2—O3^iii^	73.94 (11)		

Symmetry codes: (i) *x*, *y*−1, *z*; (ii) *x*−1, *y*−1, *z*; (iii) −*x*+1, −*y*+1, −*z*; (iv) −*x*+1, −*y*, −*z*+1; (v) −*x*+1, −*y*+1, −*z*+1; (vi) *x*, *y*, *z*+1; (vii) −*x*+2, −*y*+1, −*z*+1.
